# An antisense transcript in the human cytomegalovirus *UL87 *gene region

**DOI:** 10.1186/1743-422X-8-515

**Published:** 2011-11-11

**Authors:** Yanping Ma, Ning Wang, Mali Li, Shuang Gao, Lin Wang, Yaohua Ji, Ying Qi, Rong He, Zhengrong Sun, Qiang Ruan

**Affiliations:** 1 Virus Laboratory, The Affiliated Shengjing Hospital, China Medical University, 110004 Shenyang, Liaoning of China, China; 2 Clinical Genetics Department, The Affiliated Shengjing Hospital, China Medical University, 110004 Shenyang, Liaoning of China. China

**Keywords:** transcript, HCMV, UL87, antisense strand

## Abstract

**Background:**

Rapid advances in research on antisense transcripts are gradually changing our comprehension of genomic and gene expression aspects of the *Herpesviridae*. One such herpesvirus is the human cytomegalovirus (HCMV). Although transcription of the HCMV *UL87 *gene has not been specifically investigated, cDNA clones of *UL87 *antisense transcripts were found in HCMV cDNA libraries previously. In this study, the transcription of the *UL87 *antisense strand was investigated in three clinically isolated HCMV strains.

**Results:**

First, an 800 nucleotides transcript having an antisense orientation to the *UL87 *gene was found in a late HCMV cDNA library. Then, the *UL87 *antisense transcript was confirmed by Rapid amplification of cDNA ends (RACE) and Northern blot in three HCMV clinical strains. Two ORFs were predicted in the antisense transcript. The putative protein of ORF 1 showed a high degree of conservation among HCMV and other CMV strains.

**Conclusion:**

An 800nt antisense transcript in the *UL87 *gene region exists in HCMV clinical strains.

## Background

Human cytomegalovirus (HCMV) is the prototypical member of the subfamily *Betaherpesvirinae*. Seroepidemiologic studies have shown that the virus is widespread in the human population [1-5]. Like other herpesviruses, HCMV can not be completely eliminated by the immune system and remains either as a low-level persistent infection or in a quiescent, latent state for the lifetime of the infected person. HCMV infection is asymptomatic in most healthy adults, but causes life-threatening disease in immunologically immature or compromised individuals, including neonates [6,7], AIDS patients [8], and allogeneic transplant recipients[9].

Although the entire sequences of some HCMV strains are available (GenBank: X17403.1, FJ616285.1, GQ466044.1, GU179001, GU937742, and others), the precise number and nature of the viral genes and gene products are still in question. To date, most HCMV genes have not been extensively characterized with respect to their expression patterns. A remarkable accumulation of antisense (AS) transcripts during HCMV infection, reported by Zhang et al. [10], suggests that currently available genomic maps based on open reading frame (ORF) and other *in silico *analyses may drastically underestimate the true complexity of viral gene products.

*UL87 *is one of the 208 ORFs of the HCMV AD169 strain (GenBank: X17403.1) predicted by Chee in 1990 [11], and was reevaluated to have coding potential by Murphy [12]. Although *UL87 *was identified to encode an early protein expressed during infection with HCMV recombinant virus [13], its transcriptional pattern has not been described. However, two AS transcripts overlapping the *UL87 *gene were obtained by screening a HCMV cDNA library made during late infection, in the study by Zhang et al. [10]. Moreover, we also found two cDNA clones in a late HCMV cDNA library containing the sequence of the *UL87 *AS strand [14].

In the present study, the HCMV *UL87 *AS transcript was screened further in a late HCMV cDNA library. The structure of the *UL87 *AS transcript was investigated by RACE experiment and Northern blot in three HCMV clinical strains. An unspliced AS transcript of the *UL87 *gene was identified.

## Results

### AS transcripts in the *UL87 *region identified from the HCMV cDNA library

**Figure 1:**
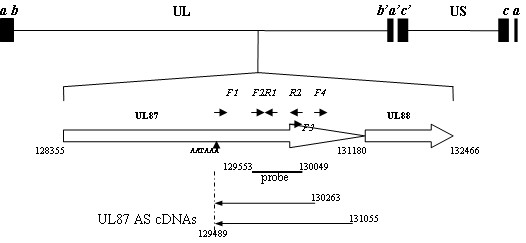
**Graphic representation of the HCMV genome, relative positions of the *UL87 *gene, *UL87 *AS cDNAs, and the primers and probe used**. Relative position is in reference to the AD19 strain (GeneBank: X17403.1).

Nineteen cDNA clones were identified as having sequences congruent with the *UL87 *gene region by graded PCR from the library. All of the 19 sequences possessed a poly(A) tail which was not coded by the HCMV genome, and were found to be homologous to the complementary strand of the *UL87 *gene. The 5' end of one of the 19 sequences was located at nt 131055, and the 5' ends of 17 other sequences were located at nt 130263. One other sequence, with a 5' end at nt 130261, was most likely a truncated cDNA created during library preparation. The 3' ends of the 19 sequences were all located at nt 129489-129491 downstream of a poly(A) signal (AATAAA) located at nt 129565-129570 (Figure 1). The sequencing results for the cDNA clones suggested that the transcripts present in the library correspond to the AS orientation of the *UL87 *gene, of which an 800 nt unspliced transcript was the dominant transcript.

### 3' and 5' ends of *UL87 *AS transcripts obtained by RACE analysis

**Figure 2:**
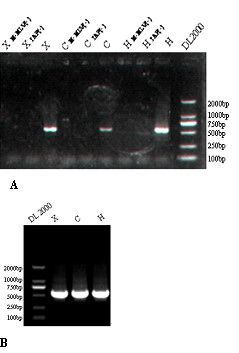
**The 5' RACE (A) and 3' RACE (B) results for the *UL87 *antisense transcript**. All RACE experiments were performed using L class RNA of the three clinically isolated strains (X, CH, H).

To confirm the existence of the *UL87 *AS transcripts, and to find other potential forms of *UL87 *AS transcripts, both 5' and 3'RACE analyses were employed with late class RNAs of the three HCMV strains (X, CH, H). The products of 3' RACE for all three strains showed an accordant band of about 500 bp (Figure 2). Sequencing results demonstrated that the 3' ends of the *UL87 *AS transcripts of all three strains were located at nt 129489-129491 downstream from a consensus poly(A) signal at nt position 129465-129470, which was identical to those of the transcripts derived from the cDNA library.

**Figure 3:**
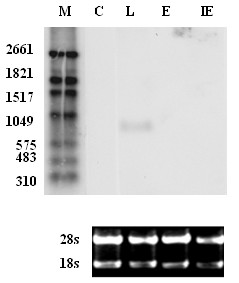
**Northern blot analysis of the *UL87 *AS transcript from the H strain**. C: mock-infected HELF cells. M: the digoxigenin-labeled RNA molecular weight marker I, which was run synchronously with the RNAs being investigated. To ensure that equal amounts of RNA were loaded, 28S and 18S rRNAs stained by EB were also included in the agarose gel.

First, 5' RACE experiments were performed using F1 and F2 (nested) primers (Table 1 Figure 1). An ~500 bp product was found in all three strains (Figure 3). The sequences from most of the clones of the 5' RACE products initiated at nt position 130267, which was four nucleotides upstream of the 5' end at nt 130263 of the transcript represented in the cDNA library. Two other clones of the 5' RACE product, of the CH strain, initiated at nt positions 130264 and 130265, respectively.

**Table 1:** Primers used in the present study

Primers		positions of 5'end ^@^	Sequences of primers (5'-3')
3' adaptor outer primer			TACCGTCGTTCCACTAGTGATTT

3' adaptor inner primer			CGCGGATCCTCCACTAGTGATTTCACTATAGG

5' adaptor outer primer			CATGGCTACATGCTGACAGCCTA

5' adaptor inner primer			CGCGGATCCACAGCCTACTGATGATCAGTCGATG

M13F			GTTTTCCCAGTCACGAC

M13R			CAGGAAACAGCTATGAC

F1	UL87 AS 5' RACE primers	129553	GAGAACCCGACCCGTAAA
		
F2		129806	GTCGGAGACGGGAGAAG AGG
		
F3		130459	GTTCAAAGCGGCTACGGCCAT
		
F4		130029	AACGCTTTCAACACCAACCGCG

R1	UL87 AS 3' RACE primers	129829	CTCCTCTTCTCCCGTCTCC
		
R2		130049	GCGGTTGGTGTTGAAAGC

Then, in order to confirm the 5'end at nt 131055 obtained in the cDNA library, two other nested primers (F3 and F4) (Table 1 Figure 1) were used. Multiple 5' ends were found, ranging from nt 130645 to nt 131430 in the three strains. However, no accordant results were found among the three strains. Moreover, the 5' end at nt 131055 could not be validated in any of the strains. The result suggested that complex structures may exist in the 5' end of the transcript.

### *UL87 *AS transcripts confirmed by Northern blot

Northern blot analysis was performed using total cellular RNAs harvested from HELF cells infected with HCMV H strain (immediate early, early, and late class), and the total RNA of mock-infected cells was used as control. RNAs were hybridized to a riboprobe (nt 129553 to 130049) complementary to the *UL87 *AS region. An 800 nt transcript was detected in late class RNA from HCMV-infected HELF cells, but not in mock-infected HELF cells (Figure 3). This suggests that the 800 nucleotide transcript is an UL87 AS transcript expressed by HCMV.

### Sequence analysis of the 800 nt *UL87 *AS transcript

**Figure 4:**
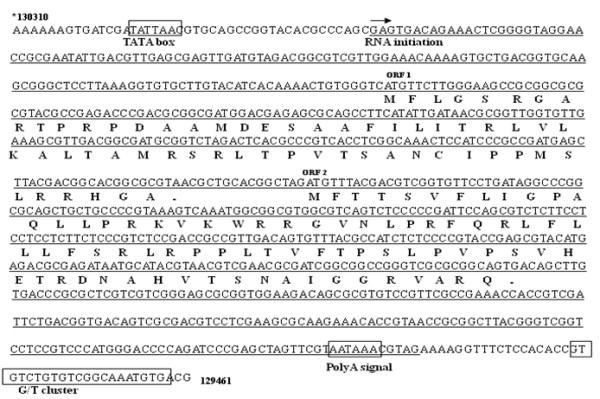
**Analyses of the 800nt *UL87 *AS transcript**. Map of the area showing the transcript (underlined) and the predicted ORF1 and ORF2. *, Position relative to AD169 strain (GeneBank: X17403.1).

The spatial location of the 800 nt *UL87 *AS transcript in the HCMV genome, as well as the primers and riboprobe used, are shown in Figure 1. The sequence of the area in HCMV AD169 is detailed in Figure 4A. A non-conventional potential TATA promoter element (TATTA) is present at 28 bp upstream of the RNA initiation site, according to sequence data obtained through 5' RACE. Besides a consensus poly(A) signal (129465-129470) located upstream, the 3' terminus, a weak consensus G/T cluster (GTGTCTGTGTCGGCAAATGTG, 129484-129464) was found downstream of the 3' terminus, an element essential for cleavage of the 3'end of the mRNAs [15,16].

Two open reading frames (ORF1 and ORF2) were predicted in the transcript, which have the potential to code for a 60-amino-acid and a 78-amino-acid protein, respectively. Prosite motif research showed that there is one N-myristoylation site and one Casein kinase II phosphorylation site in both the predicted proteins, and two Protein kinase C phosphorylation sites in the predicted protein encoded by ORF 1.

**Figure 5:**
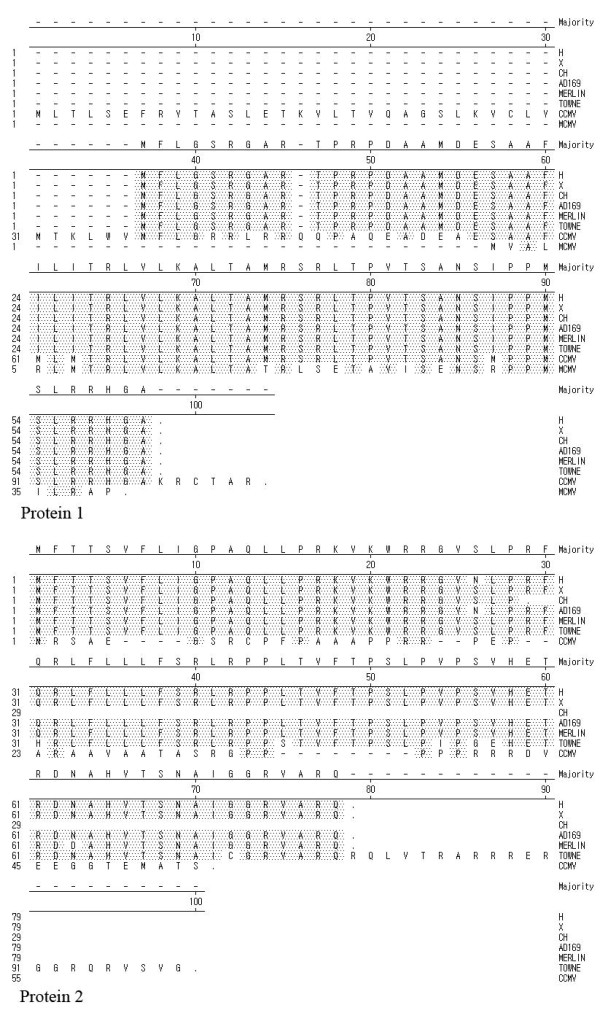
**Alignments of the putative *UL87 *AS protein 1 and protein 2 from several CMV genomes**. Amino acid alignments were done using MegAlign, from DNAstar, and *Clustal W *algorithms. The genomes compared included CCMV, MCMV, HCMV AD169, Merlin, Towne, and the three clinical strains from this study.

To study how conserved the putative *UL87 *AS proteins are among HCMV and other CMV genomes, a phylogenetic study was done using the *UL87 *AS homologous sequences of CCMV, MCMV, and HCMV of the AD169, Merlin, and Towne strains, along with the three clinical strains from this study. As shown in Figure 5, the putative proteins encoded by ORF 1 were completely consistent among these HCMV strains. CCMV and MCMV also have a similar ORF to the ORF1 of HCMV, in the same region, with the main differences located at the amino termini. The amino acid sequence of CCMV had higher homology to that of HCMV than MCMV. The ORF2 was absent in MCMV. The amino acid alignment of ORF2 did not show a high degree of conservation, in contrast to that of ORF 1, between HCMV and CCMV. Even in HCMV strains, besides amino acid changes, mutations in the termination site could be found in the CH and Towne strains (Figure 5).

## Discussion

In this study, the transcription of the AS strand of the HCMV *UL87 *gene area was investigated, and an 800 nt *UL87 *AS transcript was deeply characterized, which has been found as a cDNA clone in a late HCMV cDNA library [14]. The transcript was identified in three HCMV clinical strains.

In the present study, several lines of evidence demonstrated that an ~800 nt unspliced *UL87 *AS transcript existed among late-class transcripts during HCMV infection. An additional poly(A) tail, which was not coded by the genome, was found at the end of the *UL87 *AS transcript by sequencing the cDNA clones and 3' RACE products, confirming that it was indeed polyadenylated. The potential TATA promoter element, the consensus poly(A) signal, and the weak consensus G/T cluster all provided evidence that the novel transcript was a conventional mRNA, which could potentially encode a protein.

Two small ORFs were predicted in the transcript, which could encode proteins of 60 amino-acids and 78 amino-acids, respectively. Amino-acid sequence alignments showed that the putative protein of ORF 1 displayed highly conservation among the HCMV, CCMV, and MCMV strains. It seems likely that ORF 1 could have a protein-coding function. However, the two ORFs were predicted neither in the preliminary analysis of the HCMV genome by Chee et al. [11] nor in the re-analyses of the HCMV genome [12,17]. This is because in these analyses the authors required that any putative coding ORF encode a polypeptide of at least 100 or 80 amino acids in length. It will be important to ascertain whether the two putative proteins are in fact present in infected cells. Such studies are ongoing.

About 1.5 kb unspliced cDNA of *UL87 *AS transcripts was found in the HCMV cDNA library. Two other spliced AS transcripts expressed from the *UL87 *area have also been obtained by screening a late HCMV cDNA library [10]. Compared with the transcripts identified in the present study, they have different initiation sites (nt 134496 and 132114) but the same termination site. These results indicated that more than one transcript is expressed from the *UL87 *area in the AS orientation. There are several non-mutually exclusive explanations to account for the failure to further confirm these transcripts in this study. First, the cDNA library could not contain all of the transcripts that accumulate during infection, especially those expressed in small quantities. Second, the transcripts may vary among different strains and under different replication conditions. Third, the possible lower abundance of these transcripts in the infected HELF cells may have made detection by Northern blot difficult in this study.

The 5' RACE result with F3 and F4 as the specific nested primers did not provide an authentic identical 5' end. This could be attributed to the complicate secondary structure (hairpins or stem-loop caps) of mRNA 5'-untranslated region (5'-UTR), which may block the reverse transcription. Several RNA structures located in the 5'-UTR of eukaryotes mRNA transcripts have been shown to affect translation efficiency [18-20]. Further investigation on the 5' ends of other UL87 antisense transcripts and the secondary structure of the 5'-UTR would help to understand the characteristics of the transcript on translational regulation.

A recent study showed that a *UL87 *ORF was expressed as an early viral protein [13]. In the present study, no *UL87 *transcript was obtained when screening a HCMV cDNA library using primers located in the *UL87 *gene area. The cDNA library used in the study should contain HCMV transcripts of each infection phase, although mainly of the late class genes. However, DNA sequence analyses of several HCMV strains (AD169, GenBank: X17403.1; Merlin, GenBank: GU179001.1; Towne, GenBank: FJ616285.1) showed that the nearest poly(A) signal (AATAAA) to the 3' termini of the *UL87 *ORF was located 512 bp downstream of the *UL94 *ORF, which is about 10 kb from the 5' terminus of the *UL87 *gene. Therefore, the *UL87 *to *UL94 *genes could be co-expressed as a large polycistron. The full-length cDNA of this large transcript could not be contained in the cDNA library. Nevertheless, our study along with those of others [10,13] confirmed that both strands of the *UL87 *gene area have expression potential.

Abundant sense and antisense transcript pairs have been found by Zhang et al [10]. They obtained direct evidence for the existence of S-AS transcript pairs derived from 38 known or predicted viral genes. Individual AS transcripts have also been described for many herpesviruses, including the betaherpesviruses [21-23], the gammaherpesviruses [24-26], and especially the alphaherpesviruses [27-40]. In fact, Carter et al have predicted that genes in AS orientation to known herpesvirus genes could be common [29]. S-AS pairs may be functionally relevant with respect to regulation between them [21], so the dynamics of the S-AS transcripts of the *UL87 *gene area, along with their relationship to each other, need to be characterized further.

## Conclusion

In this study, an 800 nt unspliced *UL87 *AS transcript was identified to express in HCMV late infection phase, and two ORFs were predicted in the antisense transcript.

## Materials and methods

### Virus and specimens

Three HCMV clinical strains, named X, CH, and H, were isolated from urine samples from three infants less than 5 months old who had been hospitalized in Shengjing Hospital of China Medical University. The strains were passaged less than ten times in human embryonic lung fibroblast (HELF) cells, which were maintained in 1640 medium supplemented with 2% fetal calf serum, and 100 units penicillin/streptomycin at 37°C and 5% CO_2 _in a humidified incubator. HELF cells were inoculated with the three strains at a multiplicity of infection (MOI) of 3-5, respectively.

### RNA preparations

For preparation of immediate-early (IE) RNA of HCMV, the protein synthesis inhibitor cycloheximide (CHX) (Sigma, USA) (100 μl/ml) was added to the culture medium 1 hour before infection and the cells were harvested at 24 hours post-infection (hpi). For early (E) RNA, the DNA synthesis inhibitor phosphonoacetic acid (PAA) (Sigma, USA) (100 μl/ml) was added to the medium immediately after infection, and the cells were harvested at 48 hpi. Late (L) RNA and mock-infected cellular RNA were derived from infected and uninfected cells, respectively, cultured in parallel, and harvested at 96 hpi. Total RNAs were isolated from approximately 10^7 ^infected or uninfected HELF cells using TRIzol agent (Invitrogen, Carlsbad, CA). The isolated RNAs were treated with DNA-Free reagent (Ambion, Austin, USA) to remove possible contaminating DNA. The integrity and size of the isolated RNAs were analyzed by formaldehyde agarose gel electrophoresis. The quantity and purity of the RNAs were estimated by optical density value detection.

### Screening a HCMV cDNA library

A HCMV cDNA library had been constructed previously using the SMART technique (Clontech, USA) using the L RNA of HCMV H strain isolated from the urine sample of a HCMV-infected infant [14]. To select specific cDNA clones from the cDNA library by polymerase chain reaction (PCR), a graded PCR was set up as previously described [41,42].

Six thousand cDNA clones were screened by graded PCR using several pairs of primers (Table 1 Figure 1). The PCR conditions were initially denatured at 94 °C for 4 min, 30 cycles of 94 °C for 30 sec, 55 °C for 30 sec, and 72 °C for 1 min, followed by a final elongation of 72 °C for 10 min. Inserts in the selected clones were sequenced using vector primers (M13F and M13R). The screening results allowed us to obtain clones containing transcript sequences for both strands of the *UL87 *gene area.

### RACE

Rapid amplification of cDNA 3' ends (3' RACE) and 5'ends (5' RACE) experiments were performed with 3'-Full RACE Core Set Ver.2.0 and 5'-Full Race Kit (TaKaRa, Dalian, China), respectively. The L class RNA preparations for the three strains and RNA of mock infected cells were used as templates. First-strand cDNAs were synthesized with MMLV reverse transcriptase using oligo-dT-adaptor primers (3' RACE) and random 9-mer primers (5' RACE). Nested PCR amplifications were carried out using LA Taq (TaKaRa, Dalian, China) after reverse transcription. All of the primers are listed in Table 1 and Figure 1. The reactions were carried out at 94°C for 4 min, 30 cycles of 94°C for 30 sec, 55°C for 30 sec, and 72°C for 3 min, with a final extension at 72°C for 10 min. In 5' RACE experiments, two control reactions were performed in strict accordance with kit instructions: i) TAP (-), omitting tobacco acid pyrophosphorylase, ii) MMLV (-), omitting MMLV reverse transcriptase.

### Cloning and Sequencing

Products of RACE were separated by agarose gel electrophoresis. Different-sized products were purified using the DNA Purification Kit (Promega, Madison, WI, USA). Recovered PCR products were ligated into a pCR 2.1 TA vector (Invitrogen, China) with T4 ligase at 14°C, overnight. The ligation products were transformed into *E. coli *DH/5α competent cells. Ten clones of each purified PCR product were selected randomly for sequencing using the M13 primers and the ABI PRISM 3730 DNA analyzer (Applied Biosystems, Carlsbad, CA).

### Northern blot

For northern blot analysis, 2 μg per lane of IE, E, and L total RNA of the HCMV H strain and RNA from mock-infected HELF cells were subjected to denaturing agarose gel (1% [wt/vol]) electrophoresis in the presence of formaldehyde, alongside the digoxigenin-labeled RNA molecular weight marker I (Roche, Indianapolis, IN, USA). The *UL87 *AS-specific riboprobes were labeled using a DIG Northern starter kit (Roche, Indianapolis, IN, USA) according to the manufacturer's instructions. The riboprobes correspond to nucleotides 129553 to 130049 of the complementary strand of the AD169 sequence (Figure 1). The separated RNA fragments were transferred onto positively charged nylon membranes using capillary transfer. Then, the nylon membranes were baked at 80°C for 2 h followed by prehybridization for 30 min at 63 °C using the Dig EasyHyb-buffer (Roche, Indianapolis, IN, USA). After overnight hybridization at 63 °C, the membranes were washed according to the manufacturer's instructions. The hybridized probes were incubated with anti-digoxigenin conjugated to alkaline phosphatase and were then visualized with the chemiluminescence substrate CDP-Star (Roche, Indianapolis, IN, USA). The membranes were exposed using ChemiDoc™ XRS+ (Bio RAD, USA).

### BLAST search and sequence analysis

Standard nucleotide-nucleotide BLAST was performed on the NCBI website. The nucleotide positions referred to in this study are in reference to the sequence of the HCMV AD169 strain (GenBank: X17403.1). The following sequences were used for alignment analysis: HCMV AD169 strain (GenBank: X17403.1), Merlin strain (GenBank: GU179001.1), Towne strain (GenBank: FJ616285.1), the three clinical strains (H, CH, and X) in this study, Chimpanzee cytomegalovirus (CCMV, GenBank: AF480884), and Murine cytomegalovirus (MCMV, GenBank: AM886412). DNA alignment was done by MegAlign using Clustal W algorithms. ORFs of identified transcripts were predicted by Editseq of the DNAstar package. The motifs in the predicted proteins were predicted using GeneDoc program.

## List of abbreviations

**HCMV**: human cytomegalovirus; **ORF**: open reading frame; **HELF**: Human embryonic lung fibroblast; **MOI**: multiplicity of infection; **IE**: immediate early; **E**: early; **L**: late; **S**: sense; **AS**: antisense; **RACE**: rapid amplification of cDNA ends; **CHX**: cycloheximide; **PAA**: phosphonoacetic acid; **CCMV**: Chimpanzee cytomegalovirus; **MCMV**: Murine cytomegalovirus

## Competing interests

The authors declare that they have no competing interests.

## Authors' contributions

YPM carried out the molecular genetics studies, participated in the sequence alignment, and drafted the manuscript. NW and MLL carried out virus preparation and cell culture. LW and SG carried out the Northern blot analysis. YQ and RH carried out RNA isolation and mRNA purification. ZRS and YHJ carried out plasmid construction. As the corresponding author, QR conceived the experimental design and participated in revising the manuscript. All authors read and approved the final manuscript.

## References

[B1] Alderete JP Jarrahian S Geballe AP Translational effects of mutations and polymorphisms in a repressive upstream open reading frame of the human cytomegalovirus UL4 gene J Virol 1999 73 10 8330 8337 10482583 10.1128/jvi.73.10.8330-8337.1999 PMC112850

[B2] Adam BL Jervey TY Kohler CP Wright GL Nelson JA Stenberg RM The human cytomegalovirus UL98 gene transcription unit overlaps with the pp28 true late gene (UL99) and encodes a 58-kilodalton early protein J Virol 1995 69 9 5304 5310 7636973 10.1128/jvi.69.9.5304-5310.1995 PMC189368

[B3] Adjei AA Armah HB Gbagbo F Boamah I Adu-Gyamfi C Asare I Seroprevalence of HHV-8, CMV, and EBV among the general population in Ghana, West Africa BMC Infect Dis 2008 8 111 10.1186/1471-2334-8-111 18706107 PMC2528010

[B4] Bergallo M Costa C Terlizzi ME Margio S Sidoti F Astegiano S Sinesi F Cavallo R Reverse transcriptase-polymerase chain reaction to evaluate human cytomegalovirus lytic gene expression Mol Biotechnol 2008 40 2 144 150 10.1007/s12033-008-9070-7 18516703

[B5] Seale H MacIntyre CR Gidding HF Backhouse JL Dwyer DE Gilbert L National serosurvey of cytomegalovirus in Australia Clin Vaccine Immunol 2006 13 11 1181 1184 10.1128/CVI.00203-06 16957061 PMC1656547

[B6] Halwachs-Baumann G Genser B Pailer S Engele H Rosegger H Schalk A Kessler HH Truschnig-Wilders M Human cytomegalovirus load in various body fluids of congenitally infected newborns J Clin Virol 2002 25 Suppl 3 S81 87 12467781 10.1016/s1386-6532(02)00188-9

[B7] Lanari M Lazzarotto T Papa I Venturi V Bronzetti G Guerra B Faldella G Corvaglia L Picchio FM Landini MP Neonatal aortic arch thrombosis as a result of congenital cytomegalovirus infection Pediatrics 2001 108 6 E114 10.1542/peds.108.6.e114 11731641

[B8] Goffard A Gault E Rozenberg F Moret N Hober D Dény P Comparative sequence analysis of US28 gene of human cytomegalovirus strains isolated from HIV-positive patients Virus Genes 2006 33 2 175 181 10.1007/s11262-005-0054-4 16972032

[B9] Sacre K Nguyen S Deback C Carcelain G Vernant JP Leblond V Autran B Dhedin N Expansion of human cytomegalovirus (HCMV) immediate-early 1-specific CD8+ T cells and control of HCMV replication after allogeneic stem cell transplantation J Virol 2008 82 20 10143 10152 10.1128/JVI.00688-08 18684826 PMC2566250

[B10] Zhang G Raghavan B Kotur M Cheatham J Sedmak D Cook C Waldman J Trgovcich J Antisense transcription in the human cytomegalovirus transcriptome J Virol 2007 81 20 11267 11281 10.1128/JVI.00007-07 17686857 PMC2045512

[B11] Chee MS Bankier AT Beck S Bohni R Brown CM Cerny R Horsnell T Hutchison CA Kouzarides T Martignetti JA Analysis of the protein-coding content of the sequence of human cytomegalovirus strain AD169 Curr Top Microbiol Immunol 1990 154 125 169 2161319 10.1007/978-3-642-74980-3_6

[B12] Murphy E Rigoutsos I Shibuya T Shenk TE Reevaluation of human cytomegalovirus coding potential Proc Natl Acad Sci USA 2003 100 23 13585 13590 10.1073/pnas.1735466100 14593199 PMC263857

[B13] Isomura H Stinski MF Murata T Yamashita Y Kanda T Toyokuni S Tsurumi T The Human Cytomegalovirus Gene Products Essential for Late Viral Gene Expression Assemble into Prereplication Complexes before Viral DNA Replication J Virol 2011 85 13 6629 6644 10.1128/JVI.00384-11 21507978 PMC3126524

[B14] Ma YP Ruan Q Ji YH Wang N Li ML Qi Y He R Sun ZR Ren GW Novel transcripts of human cytomegalovirus clinical strain found by cDNA library screening Genet Mol Res 2011 10 2 566 575 10.4238/vol10-2gmr1059 21491367

[B15] Birnstiel ML Busslinger M Strub K Transcription termination and 3' processing: the end is in site! Cell 1985 41 2 349 359 10.1016/S0092-8674(85)80007-6 2580642

[B16] McDevitt MA Imperiale MJ Ali H Nevins JR Requirement of a downstream sequence for generation of a poly(A) addition site Cell 1984 37 3 993 999 10.1016/0092-8674(84)90433-1 6744418

[B17] Murphy E Yu D Grimwood J Schmutz J Dickson M Jarvis MA Hahn G Nelson JA Myers RM Shenk TE Coding potential of laboratory and clinical strains of human cytomegalovirus Proc Natl Acad Sci USA 2003 100 25 14976 14981 10.1073/pnas.2136652100 14657367 PMC299866

[B18] Babendure JR Babendure JL Ding JH Tsien RY Control of mammalian translation by mRNA structure near caps RNA 2006 12 5 851 861 10.1261/rna.2309906 16540693 PMC1440912

[B19] Kozak M Structural features in eukaryotic mRNAs that modulate the initiation of translation J Biol Chem 1991 266 30 19867 19870 1939050

[B20] Gebauer F Hentze MW Molecular mechanisms of translational control Nat Rev Mol Cell Biol 2004 5 10 827 835 10.1038/nrm1488 15459663 PMC7097087

[B21] Bego M Maciejewski J Khaiboullina S Pari G St Jeor S Characterization of an antisense transcript spanning the UL81-82 locus of human cytomegalovirus J Virol 2005 79 17 11022 11034 10.1128/JVI.79.17.11022-11034.2005 16103153 PMC1193633

[B22] Kondo K Xu J Mocarski ES Human cytomegalovirus latent gene expression in granulocyte-macrophage progenitors in culture and in seropositive individuals Proc Natl Acad Sci USA 1996 93 20 11137 11142 10.1073/pnas.93.20.11137 8855322 PMC38297

[B23] van Cleef KW Blok MJ Savelkouls KG Grauls GE Bruggeman CA Vink C Identification and characterization of two antisense transcripts from the major immediate early region of rat cytomegalovirus Arch Virol 2005 150 12 2593 2599 10.1007/s00705-005-0566-1 16052287

[B24] Prang N Wolf H Schwarzmann F Epstein-Barr virus lytic replication is controlled by posttranscriptional negative regulation of BZLF1 J Virol 1995 69 4 2644 2648 7884918 10.1128/jvi.69.4.2644-2648.1995 PMC188947

[B25] Prang N Wolf H Schwarzmann F Latency of Epstein-Barr virus is stabilized by antisense-mediated control of the viral immediate-early gene BZLF-1 J Med Virol 1999 59 4 512 519 10.1002/(SICI)1096-9071(199912)59:4<512::AID-JMV15>3.0.CO;2-B 10534735

[B26] Segouffin C Gruffat H Sergeant A Repression by RAZ of Epstein-Barr virus bZIP transcription factor EB1 is dimerization independent J Gen Virol 1996 77 Pt 7 1529 1536 8757996 10.1099/0022-1317-77-7-1529

[B27] Bohenzky RA Lagunoff M Roizman B Wagner EK Silverstein S Two overlapping transcription units which extend across the L-S junction of herpes simplex virus type 1 J Virol 1995 69 5 2889 2897 7707513 10.1128/jvi.69.5.2889-2897.1995 PMC188986

[B28] Borchers K Wolfinger U Lawrenz B Schellenbach A Ludwig H Equine herpesvirus 4 DNA in trigeminal ganglia of naturally infected horses detected by direct in situ PCR J Gen Virol 1997 78 Pt 5 1109 1114 9152430 10.1099/0022-1317-78-5-1109

[B29] Carter KL Ward PL Roizman B Characterization of the products of the U(L)43 gene of herpes simplex virus 1: potential implications for regulation of gene expression by antisense transcription J Virol 1996 70 11 7663 7668 8892886 10.1128/jvi.70.11.7663-7668.1996 PMC190835

[B30] Chang YE Menotti L Filatov F Campadelli-Fiume G Roizman B UL27.5 is a novel gamma2 gene antisense to the herpes simplex virus 1 gene encoding glycoprotein B J Virol 1998 72 7 6056 6064 9621069 10.1128/jvi.72.7.6056-6064.1998 PMC110411

[B31] Cheung AK The BamHI J fragment (0.706 to 0.737 map units) of pseudorabies virus is transcriptionally active during viral replication J Virol 1990 64 3 977 983 2154623 10.1128/jvi.64.3.977-983.1990 PMC249207

[B32] Holden VR Harty RN Yalamanchili RR O'Callaghan DJ The IR3 gene of equine herpesvirus type 1: a unique gene regulated by sequences within the intron of the immediate-early gene DNA Seq 1992 3 3 143 152 1335300 10.3109/10425179209034010

[B33] Krause PR Croen KD Straus SE Ostrove JM Detection and preliminary characterization of herpes simplex virus type 1 transcripts in latently infected human trigeminal ganglia J Virol 1988 62 12 4819 4823 2846893 10.1128/jvi.62.12.4819-4823.1988 PMC253607

[B34] Lagunoff M Roizman B Expression of a herpes simplex virus 1 open reading frame antisense to the gamma(1)34.5 gene and transcribed by an RNA 3' coterminal with the unspliced latency-associated transcript J Virol 1994 68 9 6021 6028 8057477 10.1128/jvi.68.9.6021-6028.1994 PMC237007

[B35] Li DS Pastorek J Zelník V Smith GD Ross LJ Identification of novel transcripts complementary to the Marek's disease virus homologue of the ICP4 gene of herpes simplex virus J Gen Virol 1994 75 Pt 7 1713 1722 8021600 10.1099/0022-1317-75-7-1713

[B36] Randall G Lagunoff M Roizman B Herpes simplex virus 1 open reading frames O and P are not necessary for establishment of latent infection in mice J Virol 2000 74 19 9019 9027 10.1128/JVI.74.19.9019-9027.2000 10982346 PMC102098

[B37] Voss JH Roizman B Properties of two 5'-coterminal RNAs transcribed part way and across the S component origin of DNA synthesis of the herpes simplex virus 1 genome Proc Natl Acad Sci USA 1988 85 22 8454 8458 10.1073/pnas.85.22.8454 2847162 PMC282476

[B38] Wirth UV Fraefel C Vogt B Vlcek C Paces V Schwyzer M Immediate-early RNA 2.9 and early RNA 2.6 of bovine herpesvirus 1 are 3' coterminal and encode a putative zinc finger transactivator protein J Virol 1992 66 5 2763 2772 1313901 10.1128/jvi.66.5.2763-2772.1992 PMC241032

[B39] Wirth UV Vogt B Schwyzer M The three major immediate-early transcripts of bovine herpesvirus 1 arise from two divergent and spliced transcription units J Virol 1991 65 1 195 205 1845884 10.1128/jvi.65.1.195-205.1991 PMC240505

[B40] Yamaguchi T Kaplan SL Wakenell P Schat KA Transactivation of latent Marek's disease herpesvirus genes in QT35, a quail fibroblast cell line, by herpesvirus of turkeys J Virol 2000 74 21 10176 10186 10.1128/JVI.74.21.10176-10186.2000 11024146 PMC102056

[B41] Sun Z Ren G Ma Y Wang N Ji Y Qi Y Li M He R Ruan Q Transcription pattern of UL131A-128 mRNA in clinical strains of human cytomegalovirus J Biosci 2010 35 3 365 370 10.1007/s12038-010-0041-3 20826945

[B42] Qi Y Ma Y He R Wang N Ruan Q Ji Y Li M Sun Z Ren G Characterization of 3' termini of human cytomegalovirus UL138-UL145 transcripts in a clinical strain Microbiol Immunol 2011 55 2 95 99 10.1111/j.1348-0421.2010.00294.x 21204946

